# Dynamics of end expiratory lung volume after changing positive end-expiratory pressure in acute respiratory distress syndrome patients

**DOI:** 10.1186/s13054-015-1044-0

**Published:** 2015-09-18

**Authors:** Aude Garnero, David Tuxen, Gaëlle Corno, Jacques Durand-Gasselin, Carol Hodgson, Jean-Michel Arnal

**Affiliations:** Service de réanimation polyvalente, Hôpital Sainte Musse, 54 Avenue Henri Sainte Claire Deville, 83056 Toulon, France; Australian and New Zealand Intensive Care Research Centre, Department of Epidemiology and Preventive Medicine, Monash University, The Alfred Centre, 99 Commercial Road, Melbourne, VIC 3004 Australia; Department of Intensive Care and Hyperbaric Medicine, Alfred Hospital, 55 Commercial Road, PO Box 315, Prahan, VIC 3181 Australia; Medical Research, Hamilton Medical, 8 Via Crusch, 7402 Bonaduz, Switzerland

## Abstract

**Introduction:**

Lung recruitment maneuvers followed by an individually titrated positive end-expiratory pressure (PEEP) are the key components of the open lung ventilation strategy in acute respiratory distress syndrome (ARDS). The staircase recruitment maneuver is a step-by-step increase in PEEP followed by a decremental PEEP trial. The duration of each step is usually 2 minutes without physiologic rationale.

**Methods:**

In this prospective study, we measured the dynamic end-expiratory lung volume changes (ΔEELV) during an increase and decrease in PEEP to determine the optimal duration for each step. PEEP was progressively increased from 5 to 40 cmH_2_O and then decreased from 40 to 5 cmH_2_O in steps of 5 cmH_2_O every 2.5 minutes. The dynamic of ΔEELV was measured by direct spirometry as the difference between inspiratory and expiratory tidal volumes over 2.5 minutes following each increase and decrease in PEEP. ΔEELV was separated between the expected increased volume, calculated as the product of the respiratory system compliance by the change in PEEP, and the additional volume.

**Results:**

Twenty-six early onset moderate or severe ARDS patients were included. Data are expressed as median [25th-75th quartiles]. During the increase in PEEP, the expected increased volume was achieved within 2[2-2] breaths. During the decrease in PEEP, the expected decreased volume was achieved within 1 [1–1] breath, and 95 % of the additional decreased volume was achieved within 8 [2–15] breaths. Completion of volume changes in 99 % of both increase and decrease in PEEP events required 29 breaths.

**Conclusions:**

In early ARDS, most of the ΔEELV occurs within the first minute, and change is completed within 2 minutes, following an increase or decrease in PEEP.

## Introduction

Recruitment maneuvers and positive end-expiratory pressure (PEEP) are the key components of the open lung ventilation strategy in acute respiratory distress syndrome (ARDS) [1]. Lung recruitment maneuvers aim to reaerate collapsed or non-aerated distal airways and alveoli, and PEEP prevents derecruitment to improve oxygenation and decrease the risk of ventilator-induced lung injury [2]. Applied properly and early in selected patients, lung recruitment may decrease ARDS mortality [3]. Several types of recruitment maneuvers have been described. Some use a rapid increase in pressure for a short period of time [4], whereas others use a more progressive increase in pressure. The staircase recruitment maneuver (SRM) is a step-by-step increase in PEEP with a constant driving pressure [5–8]. With each increase in PEEP, end-expiratory lung volume (EELV) increases as a result of distension of already aerated alveoli and recruitment of non-aerated lung units. However, at high levels of PEEP, there is a risk of hemodynamic compromise, especially if the step duration is prolonged. Each step length is usually 2 minutes without a strong physiologic rationale for such duration [5–8]. After the recruitment maneuver, an adequate level of PEEP is required to prevent derecruitment. The optimal PEEP setting can be determined by a decremental PEEP trial. PEEP is decreased step by step until part of the lung collapses again, which can be detected by a decrease in static compliance or transcutaneous oxygen saturation (SpO_2_) [7–10]. During the decremental PEEP trial, each step length is usually maintained for 3–5 minutes without a physiologic rationale for such duration [5–8].

The aim of the present study was to measure the dynamics of EELV changes (ΔEELV) after a step increase or decrease in PEEP (ΔPEEP) to determine the optimal duration of steps during a SRM and a decremental PEEP trial.

## Material and methods

This study was the second part of an analysis of a prospective interventional study (ClinicalTrials.gov identifier: NCT01899560) conducted between March and November 2013 in the 16-bed medical-surgical adult intensive care unit of Hôpital Sainte Musse, Toulon, France [11]. The study was approved by the local ethical review committee (Comité de Protection des Personnes Sud Méditérannée V), and informed consent was obtained from all patients or their next of kin before inclusion.

### Patients

Eligible participants were all adults 18 years of age or older who had early-onset (less than 24 h) moderate or severe ARDS according to the Berlin definition [12] and had been invasively ventilated for less than 72 h at the time of inclusion. Exclusion criteria were the following contraindications for a recruitment maneuver: bronchopleural fistula, emphysema, pneumothorax, antecedent of pneumothorax, increased intracranial pressure, pulmonary arterial hypertension with right heart failure as assessed by transthoracic echocardiography, hemodynamic instability with mean arterial pressure less than 65 mmHg, large pleural effusion as assessed by lung ultrasound, and pregnancy. Patients were mechanically ventilated using a Hamilton-S1 ventilator (Hamilton Medical, Bonaduz, Switzerland) in pressure control with 15 cmH_2_O of driving pressure, 15 breaths per minute, inspiratory/expiratory ratio of 0.33, and PEEP of 5 cmH_2_O [12]. The fraction of inspired oxygen (FiO_2_) was adjusted to target a SpO_2_ between 90 % and 94 %. A heated humidifier (MR850; Fisher & Paykel Healthcare, Auckland, New Zealand) was used for inspiratory gas conditioning. The patients were in a semirecumbent position with the head of the bed at a 45-degree angle, and they were sedated with midazolam and sufentanil to target a Richmond Agitation Sedation Scale score of −5. Cisatracurium was administered continuously [13] or in repeated injections during the procedure. The cuff of the endotracheal tube was transiently overinflated to 60 cmH_2_O before the start of the study protocol to prevent air leaks. Heart rate, invasive arterial pressure, and SpO_2_ were continuously monitored.

### Study protocol

A SRM was performed as follows: PEEP was increased from 5 cmH_2_O to 40 cmH_2_O and then decreased from 40 cmH_2_O to 5 cmH_2_O in steps of 5 cmH_2_O every 2.5 minutes. At the end of each step, airway pressure at end inspiration (P_PLAT_) and end expiration (PEEP_TOT_) was measured using a 5-second end-inspiratory and end-expiratory occlusion, respectively. Respiratory system compliance (C_RS_) was calculated as the ratio between tidal volume (V_T_) and the difference between P_PLAT_ and PEEP_TOT_: V_T_/(P_PLAT_ − PEEP_TOT_). The increase in PEEP was stopped, and the decrease was started at any level of PEEP if the patient developed bradycardia less than 60 beats per minute (bpm), tachycardia more than 140 bpm, arrhythmia, hypotension (systolic arterial pressure <80 mmHg or mean arterial pressure [MAP] <55 mmHg), or hypoxemia (SpO_2_ <85 %).

### Measurements and calculations

Flow and airway pressure (P_AW_) were measured with a proximal pneumotachograph (linear between −120 L/min and +120 L/min with ±5 % [SD], PN279331; Hamilton Medical) positioned at the Y-piece. Volumes were integrated from flow measurements. Flow, volumes, and P_AW_ were continuously recorded using Study Recorder software (Hamilton Medical) at 50 Hz.

**Fig. 1:**
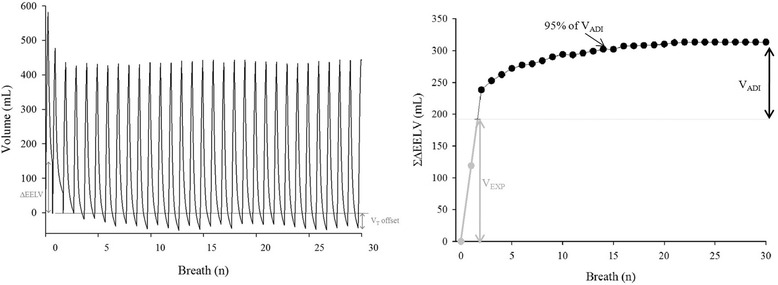
Measurement of the dynamics of the end-expiratory lung volume change (ΔEELV) after an increase in positive end-expiratory pressure (PEEP). *Left panel* displays the volume waveform for each breath following the increase in PEEP. The difference between inspired and expired tidal volumes (V_T_) was calculated and corrected by the volume offset. *Right panel* displays the dynamics of ΔEELV after the increase in PEEP. The difference between inspired and expired tidal volumes was reported breath by breath. The *gray line* is the expected increase in volume (V_EXP_) required to distend already open alveoli (calculated as the product of respiratory system compliance of the previous PEEP level by ΔPEEP), and the *black line* is the additional volume (V_ADI_). The number of breaths needed to reach 95 % of V_ADI_ was measured

ΔEELV after a PEEP step was calculated by direct spirometry, which was shown to be a robust method compared with functional residual capacity measured by a washin/washout method with insoluble gases [14]. The difference between inspiratory and expiratory V_T_ measured by direct spirometry was calculated for each breath following a PEEP step. This difference was corrected by the systematic difference, called V_T_ offset, between inspired and expired V_T_, calculated from the last 30 seconds of the step, owing to humidity and temperature differences between inhaled and exhaled gas and oxygen consumption [15]. ΔEELV was calculated as the cumulative difference between inspiratory and expiratory V_T_, corrected by V_T_ offset, measured over the 30 breaths following the PEEP step (Fig. 1). ΔEELV was partitioned between the expected volume (V_EXP_) to distend already open alveoli (calculated as the product of C_RS_ of the previous PEEP level by ΔPEEP) and the additional increased volume (V_ADI_) following the increase in PEEP. Thus, ΔEELV = V_EXP_ + V_ADI_ for each level of PEEP. During the increase in PEEP, the number of breaths required to achieve V_EXP_ to distend already open alveoli, as well as the number of breaths needed to achieve 95 % of the V_ADI_, was calculated. The number of breaths required to ensure completion of the volume change in 99 % of the increase in PEEP events was measured. During the decrease in PEEP, the number of breaths needed to achieve the expected decrease in volume (V_EXP_), as well as the number of breaths required to achieve 95 % of the additional decreased volume (V_ADD_), was calculated. Thus, ΔEELV = V_EXP_ + V_ADD_ for each level of PEEP. The number of breaths required to ensure completion of volume change in 99 % of the decrease in PEEP change events was measured.

### Statistical analysis

The distribution of the data was assessed by Kolmogorov-Smirnov test. Data with normal distribution are presented as mean±SD; others are expressed as median [25th–75th quartiles].

The numbers of breaths needed to achieve 95 % of the volume change during increase and decrease in PEEP were compared using Student’s *t* test. The number of breaths required to achieve 95 % of the additional volume according to each level of PEEP was tested using analysis of variance at both increase and decrease in PEEP. Statistic analyses were performed using SigmaStat version 3.5 and SigmaPlot version 11.0 software (Systat Software, San Jose, CA, USA).

## Results

Twenty-six patients were analyzed. The characteristics of the population at inclusion and patient outcomes are presented in Table 1.

**Table 1:** Characteristics of the population at inclusion and patient outcomes

Characteristics	Data
Number of patients	26
Sex (M/F)	19/7
Age (yr)	66±15
SAPS II	59±15
Predicted body weight (kg)	65±8
Direct ARDS, n (%)	23 (88 %)
Pneumonia, n	8
Aspiration of gastric contents, n	15
Indirect ARDS, n (%)	3 (12 %)
Pancreatitis, n	1
Sepsis, n	2
PaO_2_/FiO_2_ (mmHg)	116±37
Static compliance (ml/cmH_2_O)	37±13
Duration of invasive ventilation before inclusion (h)	19±12
Total duration of invasive ventilation (days)	10±7
Intensive care unit mortality, n (%)	8 (30 %)

*Abbreviations: ARDS* acute respiratory distress syndrome, *FiO*_*2*_ fraction of inspired oxygen, *PaO*_*2*_ partial pressure of oxygen, *SAPS* Simplified Acute Physiology Score

M/F: male/female, yr: years, kg: kilogram, n: number, %: percentage, mmHg: millimeter of mercury, ml: milliliter, cmH_2_O: centimeter of water, h: hours

The increase in PEEP was stopped prematurely in eight patients because of hypotension with MAP less than 55 mmHg (one patient at 25 cmH_2_O, two patients at 30 cmH_2_O, and five patients at 35 cmH_2_O). Totals of 170 increases in PEEP and 170 decreases in PEEP were analyzed. Totals of 17 increases in PEEP and 11 decreases in PEEP were excluded owing to technical errors. Therefore, 153 increases in PEEP and 159 decreases in PEEP were reported.

**Fig. 2:**
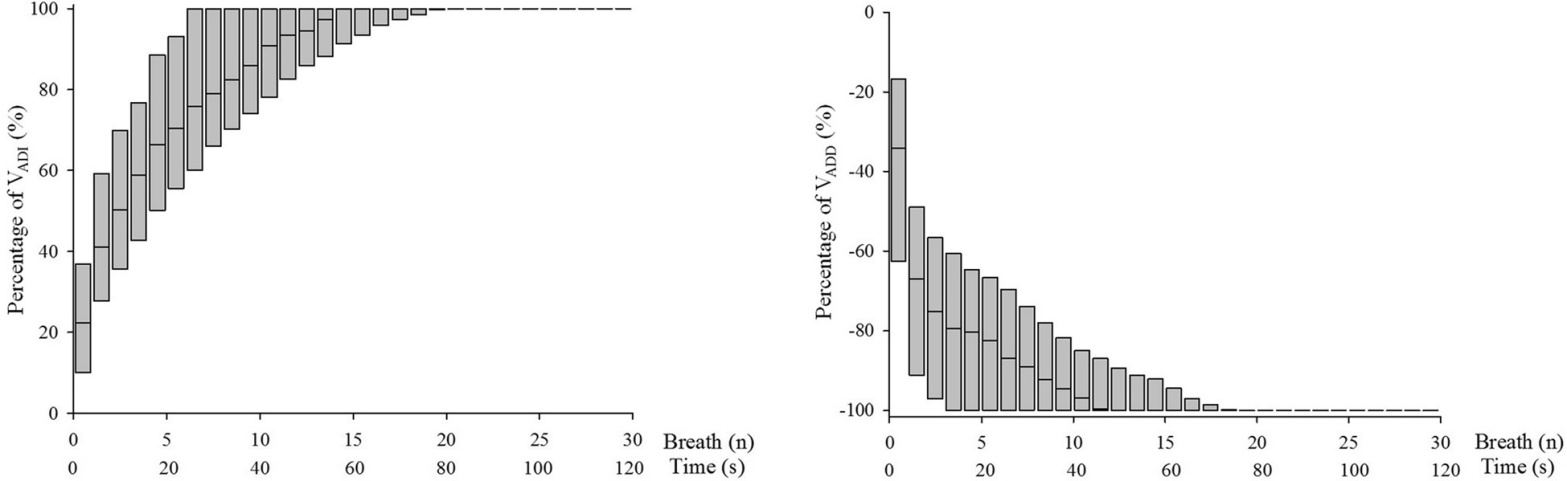
Percentage of additional volume achieved according to the number of breaths. Box plot shows medians (25th–75th quartiles) for all patients at all positive end-expiratory (PEEP) levels. *Left* and *right panels* depict increases and decreases of PEEP, respectively. *V*_*ADD*_ additional decreased volume, *V*_*ADI*_ additional increased volume

**Fig. 3:**
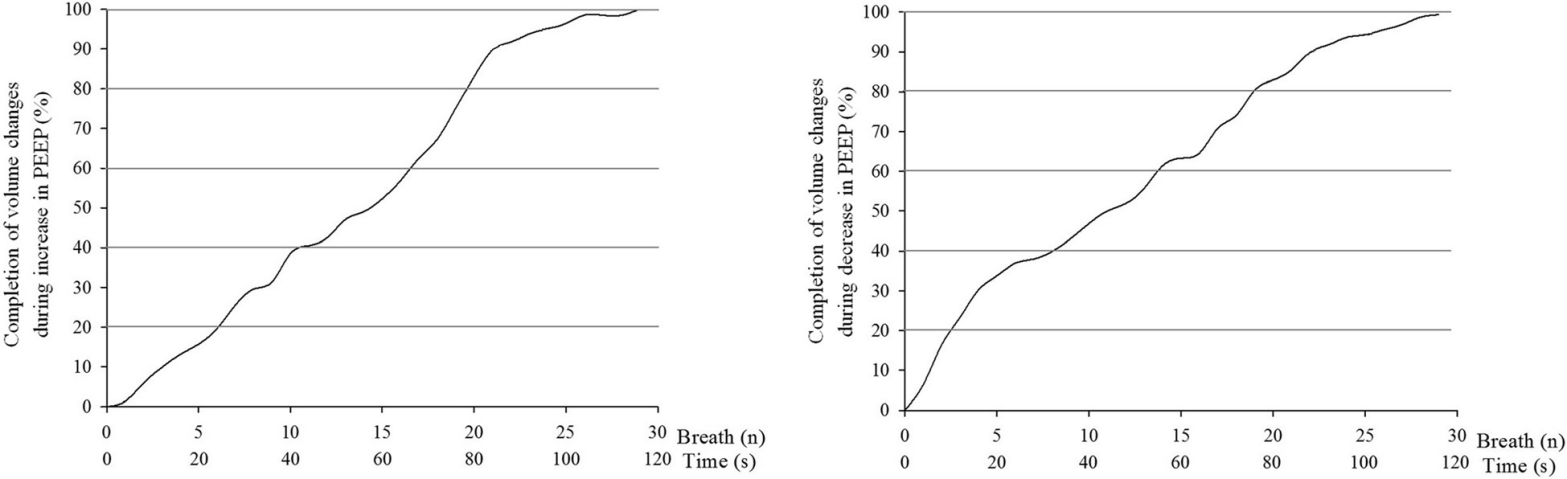
Percentage of patients’ positive end-expiratory pressure (PEEP) events that had completed volume change against the number of breaths needed to complete volume change. *Left panel:* Percentage of patients’ PEEP increase events that had completed volume change plotted against the number of breaths required to complete volume change. *Right panel:* Percentage of patients’ PEEP decrease events that had completed volume change plotted against the number of breaths needed to complete volume change

During the increase in PEEP, V_EXP_ to distend already open alveoli was achieved within 2 [2] breaths. Ninety-five percent of the V_ADI_ was achieved within 13 [6–16] breaths (52 [24–64] s) (Fig. 2). Detailed results are presented in Tables 2 and 3. During the increase in PEEP, the dynamics of ΔEELV were the same at all tested PEEP levels (*p* = 0.825) and were not correlated with V_T_ (*r*^2^ = 0.002) or PaO_2_/FiO_2_ ratio on admission (*r*^2^ = 0.170). Completion of volume change in 99 % of the patients’ PEEP increase events required 29 breaths (1 min, 56 s) (Fig. 3).

**Table 2:** ΔEELV, V_EXP_, V_AD_, and V_AD_/ΔEELV ratios for each PEEP step when PEEP was increased and decreased

PEEP steps (s)	ΔEELV (ml)	V_EXP_ (ml)	V_ADI_ (ml)	V_ADI_/ΔEELV (%)
Increase in PEEP				
5–10	309±124	193±70	116±81	34±18
10–15	340±134	196±63	144±108	37±20
15–20	328±178	181±55	147±140	35±23
20–25	297±138	174±44	123±117	36±17
25–30	290±148	142±37	154±140	45±23
30–35	211±93	129±34	82±76	33±19
35–40	203±102	111±27	89±98	34±43
Mean±SD	282±54	161±33	122±28	36±4
Decrease in PEEP				
40–35	182±133	104±31	78±114	30±22
35–30	200±111	120±31	80±99	33±18
30–25	218±72	142±41	76±45	33±12
25–20	341±120	169±55	172±119	46±18
20–15	384±174	203±56	181±140	43±12
15- 10	363±111	232±61	131±71	35±12
10–5	384±177	226±78	158±122	35±19
Mean±SD	296±92	171±51	125±47	36±6

*Abbreviations: EELV* end-expiratory lung volume, *PEEP* positive end-expiratory pressure, *V*_*ADI*_ additional increased volume, *V*_*EXP*_ expected volume

Numbers are the mean±SD of all patients

**Table 3:** ΔEELV and V_AD_ at 1 and 2 minutes and ratio between 1 and 2 minutes during increase and decrease in PEEP

PEEP step (s)	ΔEELV at 1 min (ml)	ΔEELV total (ml)	ΔEELV at 1 min/ΔEELV total (%)	V_ADI_ at 1 min (ml)	V_ADI_ total (ml)	V_ADI_ at 1 min/V_ADI_ total (%)
Increase in PEEP						
5–10	299±118	309±124	97±4	106±72	116±81	92±14
10–15	326±127	340±134	96±4	131±97	144±108	92±9
15–20	319±166	328±178	98±3	138±127	147±140	96±5
20–25	284±124	297±138	97±5	110±102	123±117	91±11
25–30	275±130	290±148	96±5	139±123	154±1140	92±11
30–35	196±82	211±93	94±6	68±68	82±76	81±25
35–40	183±77	203±102	90±8	72±71	89±98	50±35
Mean±SD	269±57	282±54	96±3	109±30	122±28	85±16
Decrease in PEEP						
40–35	174±129	182±133	97±5	70±111	78±114	92±14
35–30	196±102	200±111	99±4	75±90	80±99	100±13
30–25	216±71	218±72	99±6	74±44	76±45	98±28
25–20	329±111	341±120	97±4	160±110	172±119	95±7
20–15	363±152	384±174	96±6	160±115	181±140	91±12
15–10	358±108	363±111	99±3	126±69	131±71	96±8
10–5	373±175	384±177	97±6	150±114	158±122	89±23
Mean±SD	287±88	296±92	98±1	117±42	125±47	94±4

*Abbreviations: EELV* end-expiratory lung volume, *V*_*ADD*_ additional decreased volume, *V*_*ADI*_ additional increased volume

During the decrease in PEEP, the expected decrease in volume was achieved within 1 [1] breath. Ninety-five percent of the V_ADD_ was achieved within 8 [2–15] breaths (32 [8–60] s) (Fig. 2). Detailed results are presented in Tables 2 and 3. During the decrease in PEEP, the dynamic ΔEELV remained the same at all tested PEEP levels (*p* = 0.114) and were not correlated with V_T_ (*r*^2^ = 0.014) or PaO_2_/FiO_2_ ratio on admission (*r*^2^ = 0.002). Completion of volume change in 99 % of the patient’s PEEP decrease events required 29 breaths (1 min, 56 s) (Fig. 3).

**Fig. 4:**
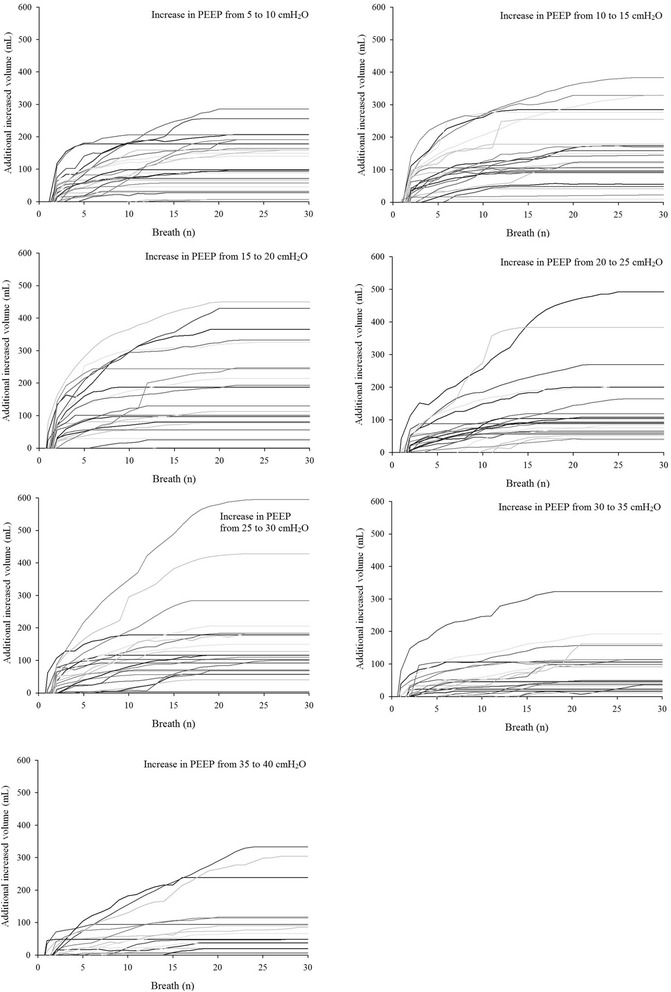
Individual additional increased volume after increase in positive end-expiratory pressure (PEEP). The expected increase in volume was subtracted

**Fig. 5:**
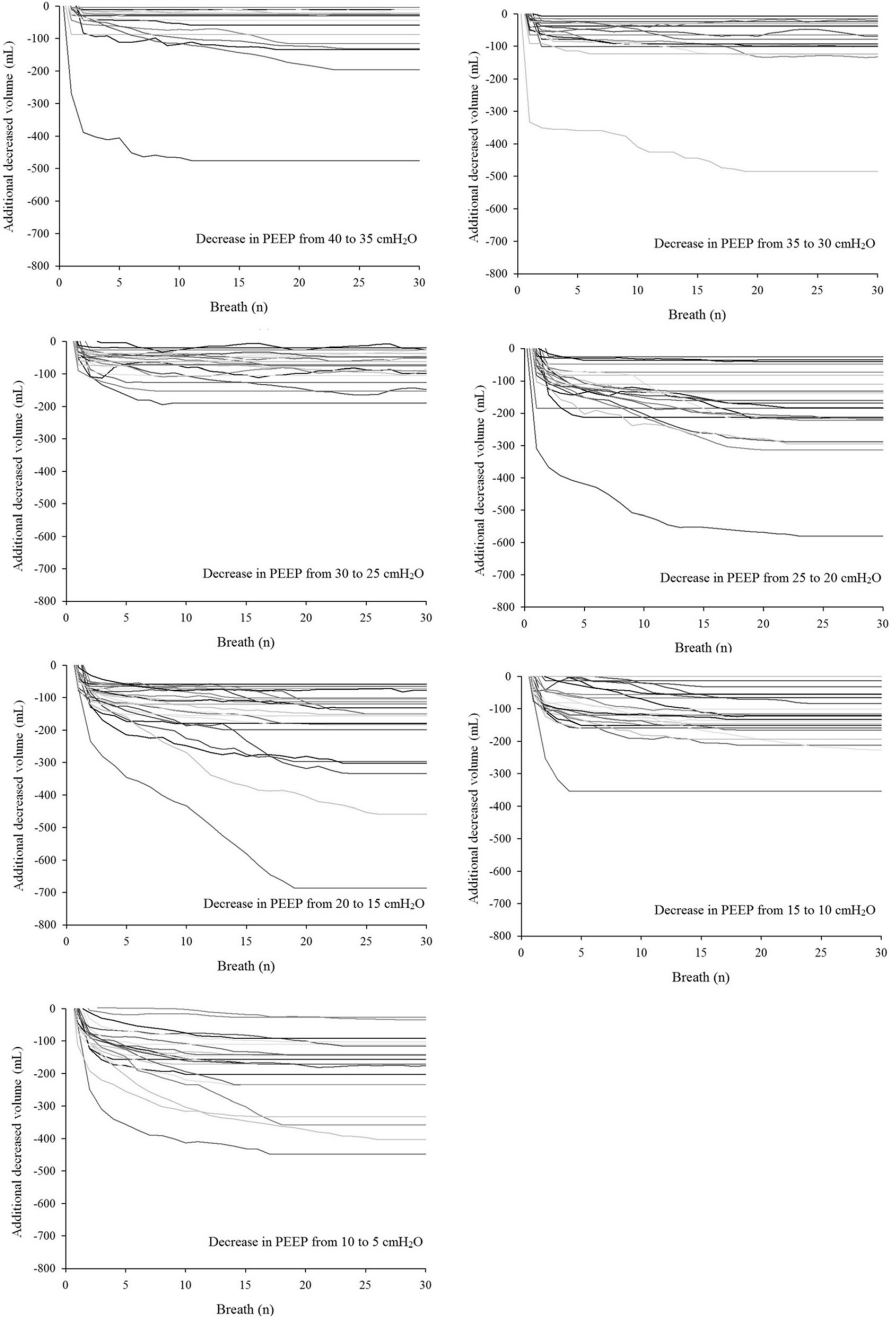
Individual additional decreased volume after decrease in positive end-expiratory pressure (PEEP). The expected decrease in volume was subtracted

For completeness, the individual additional increased and decreased volumes are presented in Figs. 4 and 5, respectively. As shown, the single patient behavior is consistent with the median values in the majority of cases.

The number of breaths needed to reach 95 % of the V_ADI_ was higher than the number of breaths required to reach 95 % of the V_ADD_ (*p* = 0.003).

## Discussion

In the present study, most of the ΔEELV during increase or decrease in PEEP occurred within the first minute and required 2 minutes to be completed. Change in 95 % of the additional volume during an increase in PEEP required more breaths than during the decrease in PEEP. If V_ADI_ is assumed to be recruited volume following the increase in PEEP and V_ADD_ is assumed to be derecruited volume following the decrease in PEEP, these results demonstrate a longer time course for 95 % recruitment compared with 95 % derecruitment but the same time course (1 min, 56 s) for completion of volume change after a PEEP step. This supports the use of a 2-minute step following both PEEP increases and decreases during a SRM if completion of volume change after each step is sought. A shorter time after PEEP changes (1 min) could be used if 95 % of volume change in 75 % of PEEP change events is an acceptable endpoint.

In the present study, 95 % of V_ADI_ was achieved within 13 [6–16] breaths after an increase of PEEP. Katz et al. found that, in patients with mild ARDS, 90 % of ΔEELV was achieved after 4.6±1.4 breaths [14]. Lung recruitment depends on applied pressure - namely, the transpulmonary pressure - and time [16]. To compare different studies and methods of recruitment, the time of exposure at high pressure needed to achieve 95 % of recruitment was calculated as the product of the number of breaths by the inspiratory time (T_I_). In this study, T_I_ was 1 second; therefore, 13 seconds of exposure at high pressure was required. In patients with severe ARDS, 14 seconds of exposure at high pressure was needed [17]. During a sustained inflation recruitment maneuver, the average time constant of the volume increase was 2.3±1.3 seconds; thus, 95 % of the recruitment occurred during the first 7 seconds [18]. The small difference between these results is due to the fact that this simple calculation considers not alveolar pressure but P_AW_. In pressure-controlled mode, alveolar pressure is equal to P_AW_ only at the end of T_I_. However, the main finding is consistent among these studies: most of the recruitment (assessed by ΔEELV) occurs rapidly during a recruitment maneuver or after a PEEP increase. Small V_ADI_ occurred beyond 1 minute up to 2 minutes after the PEEP step. Using electrical impedance tomography, it was possible to detect volume increases several minutes after the increase of PEEP [19, 20]. These changes in volume are too small to be detected by direct spirometry. Interestingly, when PEEP is increased, most of the PaO_2_ change occurs within the first 5 minutes, but PaO_2_ still increases slowly so that the equilibrium is not achieved at 1 h after the PEEP change [10]. On one hand, a possible reason could be slow alveolar recruitment, a chest wall adaptation with a shift down of the diaphragm, an improvement in cardiac output, or a change in hypoxemic vascular constriction. On the other hand, static compliance change is achieved within 5 minutes. These results are in line with the mechanism of recruitment that is time–pressure dependent and thus a progressive phenomenon of a sequence of breaking liquid bridges [21].

After a decrease in PEEP, 95 % of the V_ADD_ was achieved within 8 [2–15] breaths. Katz et al. measured that 90 % of ΔEELV in patients with mild ARDS was achieved after 3.1±0.6 breaths [14]. In the same way, the required time of exposure at PEEP needed to achieve 95 % of derecruitment was calculated as the product of the number of breaths by the expiratory time (T_E_). In this study, T_E_ was 3 seconds; therefore, 24 seconds was required. In patients with severe ARDS, 95 % of the volume change was achieved within 17 seconds of exposure at PEEP [17]. This calculation is probably overestimated because alveoli pressure reaches PEEP only at the end of T_E_. However, these results are consistent in that most of the mechanical derecruitment occurs rapidly after a decrease in PEEP. Interestingly, when PEEP is decreased, the equilibration time for arterial oxygenation is reached within 5 minutes [10]. These results support the mechanism of derecruitment as a passive phenomenon. The immediate collapse after decrease in PEEP is probably due to the gravity-dependent closure of small airways in the dependent part of the lung. Oxygenation-related variables could guide the decremental PEEP trial, but further investigations are necessary to determine the evolution of these variables during the five 5 minutes. If we consider that derecruitment increases the shunt fraction, decreases in PaO_2_ or SaO_2_ should occur rapidly after lung collapse. Conversely, static C_RS_ takes longer to decrease and should not be very informative in tracking within the minutes following the PEEP step.

The number of breaths required to recruit seems to be more than the number of breaths needed to derecruit. Recruitment is an active, progressive phenomenon of a sequence of breaking liquid bridges whereas derecruitment is a passive phenomenon due to gravity.

This study has a number of important implications. Most patients have significant recruitment after most PEEP increases, from the lowest to the highest, with no clear critical recruitment level, suggesting that all patients should be taken to the highest PEEP level (40 cmH_2_O) to maximize their recruitment. Furthermore, there was a concept that decremental PEEP was needed to find the critical PEEP level where derecruitment started to select a PEEP level at or above that level for clinical use. But again, although equivalent derecruitment did occur at lower PEEP levels, this study shows that most patients have significant derecruitment after most PEEP reductions, with no clear critical derecruitment level. This implies that the derecruitment-determined PEEP level would have to be based on a specified percentage (e.g., 10 % or 20 %) of derecruitment, possibly depending on the hemodynamic and gas exchange consequences.

The main limitation of this study is that we do not know if the additional ΔEELV is recruited volume, volume that overdistends already aerated units with a long time constant, or viscoelastance and chest wall adaptation, as we did not use any imaging of the thorax. Second, the direct spirometry method is not accurate enough to detect a ΔEELV below 10 ml. Therefore, these results may represent most but not all of the ΔEELV. Third, in an animal study, the restitution of lung volume after suctioning was significantly slower during pressure-controlled ventilation than during volume-controlled ventilation [22]. As our patients were ventilated in pressure-controlled mode, our results might have been different in volume-controlled mode. However, the breathing patterns are probably more important to explaining such differences than the mode itself. Fourth, these results concern sedated and paralyzed patients with ARDS, the population of interest in performing a SRM and decremental PEEP trial. In a spontaneously breathing patient, the time course of recruitment and derecruitment may be very different, as the inspiratory effort has a strong recruitment effect. Finally, this study included mainly ARDS caused by direct lung injury (88 %), in particular gastric aspiration. This is due to the case mix of our intensive care unit, which receives a lot of patients with coma or drug overdose complicated by gastric aspiration. Application of the results should be limited to this subgroup of the population, as the potentially recruitable lung in ARDS caused by indirect lung injury may be different [23].

In practice, these results support the use of a short duration for the PEEP step during a SRM: 1 minute to achieve most of the ΔEELV and 2 minutes to achieve complete ΔEELV. For the decreasing PEEP trial, changes in ΔEELV were even faster but difficult to assess at the bedside, so clinicians had to track oxygenation changes. For the moment, 5 minutes may be appropriate until we have data concerning the SaO_2_ changes during the first 5 minutes.

## Conclusions

In patients with early-onset moderate to severe ARDS, most of the ΔEELV occurs within the first minute and was completed within 2 minutes following an increase or decrease in PEEP. Dynamic EELV is faster after a decrease in PEEP than after an increase in PEEP. These results demonstrate that recruitment and derecruitment have different time courses and support the use of short duration (1–2 min) for the PEEP step during SRM and decremental PEEP trial.

## Key messages

- What is the key question? How long does it take to reach a new steady state in end-expiratory lung volume after an increase and a decrease in PEEP?
- What is the bottom line? Most of the change in end-expiratory lung volume occurs within the first minute, and change is completed within 2 minutes following a PEEP increase or decrease.
- Why read on? To determine the optimal duration of the PEEP step during a recruitment maneuver and decremental PEEP trial.

## Abbreviations

ARDS: acute respiratory distress syndrome
bpm: beats per minute
C_RS_: respiratory system compliance
EELV: end-expiratory lung volume
FiO_2_: fraction of inspired oxygen
MAP: mean arterial pressure
PaO_2_: partial pressure of oxygen
P_AW_: airway pressure
PEEP: positive end-expiratory pressure
PEEP_TOT_: airway pressure at end expiration
P_PLAT_: airway pressure at end inspiration
SAPS: Simplified Acute Physiology Score
SpO_2_: oxygen saturation
SRM: staircase recruitment maneuver
T_E_: expiratory time
T_I_: inspiratory time
V_ADD_: additional decreased volume
V_ADI_: additional increased volume
V_EXP_: expected volume
V_T_: tidal volume
